# What Does It Mean to Be(Come) Arctic? Functional and Genetic Traits of Arctic‐ and Temperate‐Adapted Diatoms

**DOI:** 10.1111/gcb.70137

**Published:** 2025-03-20

**Authors:** Jakob K. Giesler, Dedmer B. Van de Waal, Mridul K. Thomas, Luka Šupraha, Florian Koch, Tilmann Harder, Carla M. Pein, Uwe John, Sylke Wohlrab

**Affiliations:** ^1^ Ecological Chemistry Section, Alfred Wegener Institute Helmholtz Centre for Polar and Marine Research Bremerhaven Germany; ^2^ Department of Aquatic Ecology Netherlands Institute of Ecology (NIOO‐KNAW) Wageningen the Netherlands; ^3^ Department of Freshwater and Marine Ecology, Institute for Biodiversity and Ecosystem Dynamics University of Amsterdam Amsterdam the Netherlands; ^4^ Department. F.‐A. Forel for Environmental and Aquatic Sciences and Institute for Environmental Sciences University of Geneva Geneva Switzerland; ^5^ Department of Biosciences University of Oslo Oslo Norway; ^6^ Norwegian Institute for Water Research (NIVA) Oslo Norway; ^7^ Department of Biology and Chemistry University of Bremen Bremen Germany; ^8^ Helmholtz Institute for Functional Marine Biodiversity at the University of Oldenburg (HIFMB) Oldenburg Germany

**Keywords:** convergent evolution, photoperiod, phytoplankton, poleward migration, range shift, *Thalassiosira*

## Abstract

Climate change‐induced warming is expected to drive phytoplankton poleward as they track suitable thermal conditions. However, successful establishment in new environments requires adaptation to multiple abiotic factors beyond temperature alone. As little is known about how polar species differ in key functional and genetic traits, simple predictions of poleward movement rely on large assumptions about performance in other relevant dimensions other than thermal responses (e.g., light regime, nutrient uptake). To identify evolutionary bottlenecks of poleward range shifts, we assessed a range of thermal, resource acquisition, and genetic traits for multiple strains of the diatom *Thalassiosira rotula* from the temperate North Sea, as well as multiple strains of the closely related Arctic *Thalassiosira gravida* . We found a broader thermal range for the temperate diatoms and a mean optimum temperature of 10.3°C ± 0.8°C and 18.4°C ± 2.4°C for the Arctic and temperate diatoms, respectively, despite similar maximum growth rates. Photoperiod reaction norms had an optimum photoperiod of approximately 17 h for temperate diatoms, whereas the Arctic diatoms exhibited their highest growth performance at a photoperiod of 24 h. Nitrate uptake kinetics showed high intraspecific variation without a habitat‐specific signal. The screening for convergent amino acid substitutions (CAAS) of the studied diatom strains and other publicly available transcriptomes revealed 26 candidate genes in which potential habitat‐specific genetic adaptation occurred. The identified genes include subunits of the DNA polymerase and multiple transcription factors (zinc‐finger proteins). Our findings suggest that the thermal range of the temperate diatom would enable poleward migration, while the extreme polar photoperiods might pose a barrier to the Arctic. Additionally, the identified genetic adaptations are particularly abundant in Arctic diatoms as they may contribute to competitive advantages in polar habitats beyond those detected with our physiological assays, hampering the establishment of temperate diatoms in Arctic habitats.

## Graphical abstract

Climate change is expected to drive phytoplankton poleward, but successful establishment in new environments requires adaptation to multiple factors beyond temperature. This study compares two common temperate and Arctic diatom species, finding that while temperate diatoms have a broader thermal range suitable for poleward migration, extreme polar photoperiods might pose a barrier. Additionally, evolved genetic adaptations in Arctic diatoms may provide competitive advantages in polar habitats, potentially hindering the establishment of temperate species in Arctic environments.

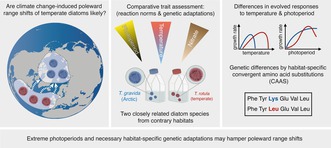

## 1 Introduction

The Arctic Ocean is among the biologically most productive areas in the world (Ardyna et al. 2013) and its resident primary producers are therefore of particular importance for the biological carbon pump and the marine food web (Tremblay et al. 2015). As the Arctic is warming several times faster than the global average (Maturilli et al. 2013; Rantanen et al. 2022), alterations in primary producer communities are likely due to both warming‐induced abundance changes in resident species (Ahme et al. 2023) as well as poleward range shifts of temperate phytoplankton species (Benedetti et al. 2021; Chivers et al. 2017; Hodapp et al. 2023). Thermal performance thus has become the prime parameter for estimating and modeling potential future species distributions (Benedetti et al. 2021; Hodapp et al. 2023). However, migrating species will encounter changes in other abiotic factors than temperature (i.e., a different selection regime), which leads to large assumptions that have to be made about the performance in other dimensions relevant for primary producers. Species distribution models project planktonic poleward range shifts at a speed of 35 km per decade (Benedetti et al. 2021), yet field data indicate that particularly diatoms encounter considerable barriers, slowing down their poleward range expansion (Chivers et al. 2017). The nature of these barriers is currently unknown but may indicate mismatches of evolved responses of temperate diatoms to latitude‐characteristic drivers prevailing in polar regions. Consequently, to address the potentials and bottlenecks of poleward range shifts of temperate phytoplankton, it is necessary to define what a comparative Arctic adaptation constitutes. Therefore, identifying habitat‐specific adaptations of Arctic primary producers that differ from those of temperate species and thus could hamper poleward range shifts provides a necessary baseline to assess future species distributions.

In addition to water temperature, relevant environmental parameters to which temperate primary producers would need to adapt in case of ongoing range extension include different photoperiods with pronounced extremes in duration and transition in the Arctic. From a biogeochemical perspective, changes in dissolved inorganic nitrogen concentration have emerged as key parameters controlling primary production in the Arctic (Juranek 2022). Previous studies resolved several molecular and physiological mechanisms which potentially provide habitat‐specific adaptations. Thermal adaptations hence involve adjustments in gene expression of several metabolic pathways and cellular processes (Liang et al. 2019) along with physiological adjustments in photosynthetic capacity (Schaum et al. 2017). Known adaptations to different photoperiods include rhythmicity in basic biological processes regulated by an endogenous circadian clock (Annunziata et al. 2019), as well as mechanisms to reduce oxidative stress on photosynthesis (Croteau et al. 2020; Hunken et al. 2008) and on the lipidome (Svenning et al. 2024). Lower C:N ratios due to higher protein contents in Arctic diatoms indicate higher cellular nitrogen demands compared to temperate relatives (Lacour et al. 2017).

However, a so far missed opportunity to study habitat‐specific genetic adaptations of phytoplankton more holistically is convergent amino acid substitutions (CAAS) at the level of protein sequences. CAAS, where the same amino acid changes occur phylogenetically independently in different lineages, indicate habitat‐specific adaptation, as the underlying substitutions likely confer a selective advantage in the respective environment (Rey et al. 2019). CAAS have already provided valuable insights into the adaptive evolution of other organisms (Bo et al. 2022; Foote et al. 2015; Hill et al. 2019), and therefore offer a compelling approach for the identification of distinctive molecular changes that facilitate phytoplankton to thrive in different habitats.

Evolutionary adaptations to habitat‐specific environmental characteristics that arise at the genomic level are reflected in species‐ and habitat‐specific reaction norms to different environmental drivers. These reaction norms allow to draw conclusions about a species adaptive history in its respective habitat. Yet, only a few thermal performance curves from polar species have been published (but see [Boyd et al. 2013; Rehder et al. 2024]) and performance curves to photoperiods or nutrient concentrations are largely missing (but see [Guérin et al. 2024]). In line with this, we currently lack comparative investigations on closely related species from temperate versus Arctic habitats (but see [Liang et al. 2019]) to account for the non‐independence of phylogeny on trait evolution (Hansen and Martins 1996).

By addressing what an Arctic versus temperate adaptation of diatoms constitutes, we aimed to identify which potential bottlenecks constrain the expansive potential of temperate species to Arctic conditions. This was implemented by comparing thermal, photoperiodic, and nitrate uptake‐related reaction norms and key functional traits of two closely related Arctic ( *Thalassiosira gravida* ) and temperate diatoms ( *Thalassiosira rotula* ). Their phylogenetic proximity, in combination with separated geographic distribution (Šupraha et al. 2022), makes these diatoms specifically suitable to compare inherent habitat‐specific traits. To extend our knowledge beyond measurable key functional traits, we assessed habitat‐specific genetic adaptations that have repeatedly been selected for in diatoms as a response to Arctic or temperate conditions (CAAS). In addition to differences in reaction norms, CAAS delivered further indications of necessary adaptations for poleward range shifts. We thus provide missing information on evolved differences in habitat‐specific adaptations that could hinder poleward shifts of temperate species, and thus a necessary baseline for assessing future species distributions.

## 2 Material and Methods

### 2.1 Biological Material and Culture Conditions

The experiments in this study comprise five Arctic‐adapted strains of *T. gravida* obtained from the Norwegian Culture Collection of Algae (NORCCA strain numbers UiO478; UiO483; UiO484; UiO447; UiO448, here called A1, A2, A3, A4, and A5) as well as five temperate‐adapted strains of *T. rotula* . This includes one strain obtained from the Harder Lab (University of Bremen; strain number S16, here called T1) isolated from Helgoland, two strains isolated from the German Bight (strain Wilhelmshaven, strain Sylt, here called T2 and T3), and two strains obtained from the Roscoff Culture Collection (strain numbers RCC‐778, RCC‐290, here called T4 and T5) isolated from the English Channel. Further details about the strains used in this study can be found in Table S1. The number of strains used differs between experiments because not all strains were available at all times during the different experimental parts of this study. *T. gravida* and *T. rotula* strains were identified by their ITS sequences based on taxonomic definitions by Whittaker et al. (2012) (see Figure S1). Cultures were maintained in climate chambers at a light intensity of 30 μmol^−2^ photons m^−2^ s^−1^ at a photoperiod of 16:8 h light: dark and a temperature of 4°C and 15°C for the Arctic and temperate strains, respectively. Cultures were kept in exponential growth by semi‐continuous dilution with K‐medium (Keller et al. 2007) modified by Si‐enrichment (1.06 × 10^−4^ M Na_2_SiO_3_ × 9 H_2_O) and no ammonium addition.

### 2.2 Temperature Performance Curves

Temperature performance curves (TPCs) were assessed for 5 Arctic strains of *T. gravida* and 5 temperate strains of *T. rotula* . The TPC assays were conducted on a thermal gradient block with a respective temperature gradient for the Arctic (−0.5°C, 2.3°C, 5.0°C, 7.3°C, 9.9°C, 12.3°C, 15°C) and temperate (2.8°C, 7.0°C, 11.0°C, 14.5°C, 18.0°C, 21.4°C, 24.5°C, 27.8°C) diatoms based on thermal sensitivities from other temperate and polar phytoplankton species (Boyd et al. 2013; Rehder et al. 2024). TPCs were conducted at a light intensity of 30 μmol photons ^2^m^−2^ s^−1^ and a light:dark cycle of 16:8 h. In order to acclimate the cultures to their experimental conditions, 200 mL of batch cultures from each strain were incubated at a cell density of 500 cells mL^−1^ at the mean temperature of the chosen gradient for the Arctic or temperate strains. Over a period of 7 days, the cultures were then gradually heated or cooled until reaching their respective final experimental temperature with a maximum heating/cooling rate of 1.8°C day^−1^. After the stock cultures reached their experimental temperatures, they were kept in the exponential growth phase for 8 more days for thermal acclimation, by semi‐continuous dilution with modified K‐medium. After this acclimation period, 4 replicates of 50 mL culture flasks (Sarstedt, Germany) were inoculated with the final experimental cultures to an initial cell density of 250 cells mL^−1^ in 40 mL of media. Sampling was conducted daily by fixing 500 μL subsamples from each experimental unit with 2% final concentrated Lugol's solution in a 48‐well microplate (Sarstedt, Germany). Samples were quantified microscopically using a Zeiss Axiovert 10C inverted light microscope. Cell densities were tracked daily and the treatments were ended upon reaching stationary phase (i.e., begin of saturation in the logistic growth curve). Raw data are deposited in the Pangaea data repository (Giesler, Wohlrab et al. 2024).

### 2.3 Photoperiod Reaction Norms

To assess the growth response to different photoperiods (1, 4, 8, 16, 24 h), photoperiod reaction norms assays were conducted using 4 Arctic *T. gravida* (A1, A2, A3, A4) and 4 temperate *T. rotula* (T1, T2, T3, T5) strains. These growth assays were conducted in nanocosm well‐plate photobioreactors, which allowed for high replication numbers, well‐specific programmed light settings, and a lab‐independent and reproducible experimental setup (Volpe et al. 2021). The experiments were conducted in modified K‐medium (see above) at a light intensity of 30 μmol photons^−2^ m^−2^ s^−1^ and an experimental temperature representing the lowest optimum temperature among the studied genotypes of Arctic and temperate strains assessed in the TPCs (i.e., 9°C for *T. gravida* and 16°C for *T. rotula*). To acclimate the cultures to their experimental conditions, 24 replicates per strain with an experimental volume of 300 μL were inoculated in 96 well‐plates at an initial chl‐α fluorescence value close to the blank value measured in the medium, except for 1 h and 4 h photoperiod treatments, which were inoculated at twice the blank value to obtain sufficient biomass. The microtiter plates were sealed with a gas‐permeable membrane (Breathe Easy, Sigma–Aldrich, USA) and placed in a climate cabinet at the respective experimental temperature. After seven days of acclimation to the experimental conditions, cultures were diluted with temperature‐acclimated medium and inoculated in a new well‐plate close to their fluorescence blank value for the actual growth experiment. This acclimation duration was chosen as it covers both short‐term and long‐term photoacclimation time scales (Lutz et al. 2001), that is, no further treatment effects on cellular chl‐α content can be assumed, and robust growth rates can directly be calculated from fluorescence intensity values. Chl‐α fluorescence intensity was measured daily at the same time after 10 min of dark acclimation using a photo‐spectrometric plate reader at an excitation wavelength of 440 nm and emission of 680 nm (ClarioStar Plus BMG Labtech for Arctic strains; Spark TECAN for temperate strains). Chl‐α fluorescence was tracked regularly, and the treatments were ended upon reaching stationary phase (i.e., begin of saturation in the logistic growth curve). Raw data are deposited in the Pangaea data repository (Giesler, Ahme et al. 2024).

### 2.4 Nitrate Uptake Assays

^15^N nitrate uptake assays were conducted by means of tracer additions of highly enriched (98%) ^15^N‐labeled nitrate (Mulholland et al. 2009, 2002) for 4 Arctic and 3 temperate strains of *T. gravida* and *T. rotula* , respectively (strains A1, A2, A3, A5 and T1, T2, T3). For precultivation, 2 L batch cultures of each strain were grown at a light intensity of 25 μmol photons^−2^ m^−2^ s^−1^ and a light:dark cycle of 16:8 h. Cultures were grown in modified ESAW medium (Harrison et al. 2008) at the lowest respective optimum temperature (9°C for Arctic strains and 16°C for temperate strains). The nitrate concentration in the medium corresponded to 1/5 K‐medium (approximately 100 μmol L^−1^) which was considered necessary to obtain sufficient biomass for the ^15^N isotope analysis. In the mid‐exponential growth phase, 90% of the supernatant of the batch culture flasks was decanted, and the sedimented cells were resuspended in nitrate (and ammonia) free modified ESAW medium. Overnight, the cells sedimented again, and the procedure was repeated two more times with a time interval of 24 h in between. This dilution led to a calculated final concentration of 0.1 μmol NaNO_3_ L^−1^ in the medium at maximum, without considering the N uptake of the diatoms in the batch cultures, which depleted the dissolved nitrate concentrations even further. This procedure was considered the best possible compromise between a sufficiently dense culture and nitrogen depletion. Moreover, it has been demonstrated that 24 h of N‐starvation were sufficient to trigger high nutrient uptake in the marine diatom *Phaeodactylum tricornutum* (Raimbault et al. 1990). Accordingly, 24 h after the last dilution step with nitrogen‐free medium, the assay was prepared by filling 40 mL culture from the batch cultures into 50 mL culture flasks for the nitrate addition treatments comprising 7 nitrate levels (*n* = 4) as well as a nitrate deplete control treatment. The chosen seven levels of final nitrate concentrations were 0.1, 0.4, 0.8, 2, 10, 50, and 100 μmol NaNO_3_ L^−1^ at a ratio of 1:1 of ^14^N:^15^N for levels ≤ 2 μmol NaNO_3_ L^−1^ and 9:1 for levels > 2 μmol NaNO_3_L^−1^. The experimental units were incubated for 40 min at the same culture conditions as the batch cultures (see above) and were then filtered onto pre‐combusted glass microfiber filters (Whatman GF/F, Maidstone, UK). After filtration of the experimental unit, another 100 mL of nitrate‐free medium was filtered to reduce dissolved ^15^N contamination on the filter. The filters were dried at 60°C for 48 h and then folded into tin capsules, which were stored in the dark until further analysis. POC/PON and the particulate ^15^N:^14^N ratio were measured using an elemental analyzer (Flash 2000, Thermo Scientific, Karlsruhe, Germany) coupled to an isotope ratio mass spectrometer (IRMS, Thermo Scientific, Karlsruhe, Germany; (Morrien et al. 2017)). Raw data are deposited in the Pangaea data repository (Giesler, Van de Waal et al. 2024).

### 2.5 Transcriptomic Profiles

To assess transcriptomic profiles, a 40 mL culture of each strain was filtered onto a 10 μm nylon filter (Merck Millipore, USA) which was fixed in 1 mL TriReagent (Sigma–Aldrich, St. Louis, MI, USA) with glass beads and immediately frozen at −80°C until further processing. Cultures were kept as described in the culture conditions section and were harvested in the mid‐exponential growth phase at the same time in the middle of the light phase. RNA extraction was conducted as described in detail by Wohlrab et al. (2017). The subsequent library preparation was conducted using the Illumina Stranded mRNA Prep Ligation Kit (Illumina, San Diego, CA, USA) following the manufacturer's protocol. The resulting paired‐end cDNA libraries were sequenced on a NextSeq 2000 (Illumina, San Diego, CA, USA) using the P3 Reagents kit (2 x 150 cycles). The data for this study have been deposited in the European Nucleotide Archive (Giesler et al. 2025).

### 2.6 Statistical Analysis of TPCs

To fit strain‐specific TPC models, maximum growth rates were determined for each experimental unit (i.e., for each strain at each experimental temperature and each replicate) using the all_splines function of the “growthrates” package, which determines the maximum growth rates from the log‐linear part of the growth curve by means of smoothing splines (Petzoldt et al. 2017). TPC models were fitted to these growth rates across the tested temperatures by means of the “rTPC” package (Padfield 2023). Precisely, the model by Thomas et al. (2017) was applied, which allows for negative growth rates (i.e., mortality) beyond the critical thermal minimum and maximum temperatures. However, we did not employ modeled growth rate predictions outside of the experimentally tested thermal gradient. Subsequently, non‐parametric bootstrapping was conducted to estimate model uncertainty and 95% confidence intervals for TPC parameters (i.e., optimum temperature [*T*_opt_], maximum growth rate [*μ*_max_], thermal breadth at 80% of *μ*_max_ [Tb_80_], and activation energy [*E*_*A*_]). To test for a general difference between Arctic and temperate populations, a Welch *t*‐test was conducted with the mean of the respective TPC parameters (see above) from each strain as the dependent variable and the population origin as the independent variable. Scripts to reproduce statistical analyses are deposited on Github (https://github.com/jakobgiesler/arctic_traits/).

### 2.7 Statistical Analysis of Photoperiod Growth Assays

After the mean blank value of the medium was subtracted from the measured chl‐α fluorescence values of the samples, the “growthrates” package (Petzoldt et al. 2017) was used to obtain *μ*_max_ values for each experimental unit. In order to specifically analyze differences in photoperiod reaction norm shape between Arctic and temperate origin, growth rates of each species were normalized by their respective highest achieved growth rates. To test for shape differences in photoperiod reaction norms, generalized additive models (GAMs) were fitted to each species' data and differences tested by assessing the significance of the difference smooth term between both species, as described in detail in Rose et al. (2012) and implemented in the R package mgcv (Wood 2006).

### 2.8 Calculation and Statistical Analysis of N‐Uptake Rates

Values for *δ* ^15^N were determined by measuring the difference in ^15^N levels between the sample and a reference gas containing 0.366 atom% ^15^N. Subsequently, these *δ* ^15^N values were employed in computing absolute nitrate uptake rates (*V*_abs_) for the tested nitrate concentrations using the mixing model of Montoya et al. (2002) and using equations from Orcutt et al. (2001). The obtained *V*_abs_ values (μmol N L^−1^ h^−1^) for the respective tested concentrations were then normalized by cell density, and the parameters of the Michaelis–Menten function were estimated:

$$V=\frac{V_{\max}\;S}{K_{s}+S}$$

where *V* (μmol N h^−1^ cell^−1^) represents the cell‐normalized nitrate uptake rate, *V*_max_ (μmol N h^−1^ cell^−1^) represents the highest achieved uptake rate, *K*_*s*_ is the half‐saturation constant (μmol L^−1^) and *S* is a given nitrate concentration in the growth medium (μmol L^−1^).

### 2.9 Analysis of Transcriptomic Data

The Illumina basecall files underwent demultiplexing and were transformed into FASTQ files using the Illumina bcl2fastq conversion tool (version 2.20). Subsequently, the FASTQ files were processed using CLC Genomics Workbench (version 21, Qiagen, Hilden) including paired‐end mapping, adapter and quality trimming, and de novo assembly into contigs with default parameters for Illumina sequence data. Open reading frames (ORFs), coding sequences, and amino acid translation were created with transdecoder (Haas 2013).

For CAAS analysis, additional transcriptomes and metatranscriptomes were obtained from the respective sources listed in Table S2. This allowed us to more confidently assess convergence due to origin and to distinguish it from speciation events, as well as to assess whether a given CAAS‐bearing transcript is expressed in the native habitat (metatranscriptome), that is, to highlight its ecological significance. In cases where raw reads or nucleotide data were retrieved, processing was as described above for the transcriptome data. Diatom‐specific amino acid sequences were selected from the metatranscriptomes by phylogenetic placements with the MMseqs2 taxonomy tool (Mirdita et al. 2021) and a custom‐built reference database containing revised sequences from the MMETSP project (Van Vlierberghe et al. 2021) as well as the *T. gravida* strains used in this study. All diatom‐originated amino acid sequences from cultures and metatranscriptomes were clustered into similarity groups with an identity threshold of 75% with Silix (Miele et al. 2011). Sequences in each cluster were aligned with MAFFT (Katoh and Standley 2013) and trimmed with the gappyout algorithm implemented in trimAI (Capella‐Gutierrez et al. 2009). Trimmed sequences were analysed for convergent amino acid substitutions using CAAStools (Barteri et al. 2023) and filtered according to the following criteria: (I) each substitution must additionally occur in a temperate and an Arctic diatom species other than *T. gravida* and *T. rotula* , and (II) each CAAS gene variant must be expressed in both the Arctic and temperate metatranscriptome collections. The position of each CAAS in each alignment was manually checked to ensure reliability, that is, to occur in an otherwise gapless conserved region. The resulting genes were annotated using eggNOG‐mapper version 2.1.12 (Cantalapiedra et al. 2021), including de novo screening for the occurrence of PFAM protein domain motifs (Mistry et al. 2020).

## 3 Results

### 3.1 Temperature Performance Curves

**FIGURE 1:**
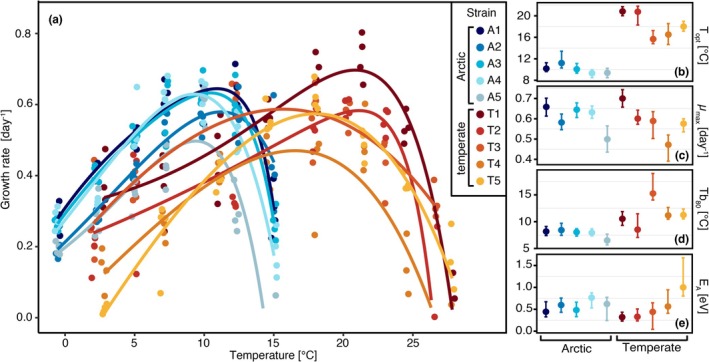
Temperature performance curves and associated parameters for *T. gravida* (strains A1–A5) and *T. rotula* (strains T1–T5) indicated by color with (a) fitted TPC models to growth rate on the *y*‐axis and temperature on the *x*‐axis; (b) optimum temperature (*T*_opt_); (c) maximum growth rate (*μ*_max_); (d) thermal breadth at 80% of *μ*_max_ (Tb_80_); and (e) activation energy (*E*_*A*_). TPC parameters (panels b–e) are given as mean ± 95% confidence intervals as error bars. For visibility, *x*‐axis jittering was added to the points.

For the Arctic strains, the fitted temperature performance curve models displayed an optimum temperature (*T*_opt_) ranging from 9.3°C to 11.2°C with an overall mean of 10.3°C ± 0.8°C (Figure 1). For the temperate strains, *T*_opt_ ranged from 15.7°C up to 20.8°C with a mean of 18.4°C ± 2.4°C. *T*_opt_ differed as a response to Arctic or temperate origin (*p* < 0.001). With regard to the maximum achieved growth rate across all tested temperatures (*μ*_max_), Arctic and temperate strains had a mean *μ*_max_ of 0.60 ± 0.07 and 0.59 ± 0.08, respectively, and showed no significant difference by origin. Furthermore, the thermal breadth was compared at a level of 80% of *μ*_max_ (Tb_80_) and revealed a significant origin‐specific difference between the studied diatom strains (Table 1) with the Arctic strains showing a narrower breadth (7.82°C ± 0.75°C) compared to the temperate strains (11.35°C ± 2.44°C). The activation energies (*E*_*A*_) of the fitted TPCs ranged from 0.32 to 1.00 eV. However, no origin‐specific differences could be detected. Overall, the thermal traits of the Arctic and temperate diatoms demonstrated differences in *T*_opt_ and Tb_80_, while no differences in *μ*_max_ and *E*_*A*_ were evident.

**TABLE 1:** Statistical results of TPC parameters. Welch *t*‐test results on differences across Arctic and temperate strain origin for the TPC parameters optimum temperature (*T*_opt_), maximum growth rate (*μ*_max_), thermal breadth at 80% of *μ*_max_ and activation energy (*E*_*A*_). *T* and *p*‐values are reported for each effect.

Parameter	df	Arctic versus temperate	
		*t*	*p*
*T*_opt_	4.8	−7.443	< 0.001*
*μ*_max_	7.6	0.335	0.747
Tb_80_	4.8	−3.086	0.029*
*E*_*A*_	5.6	0.369	0.726

* *p* < 0.05; indicate significant effects.

### 3.2 Photoperiod Reaction Norms

**FIGURE 2:**
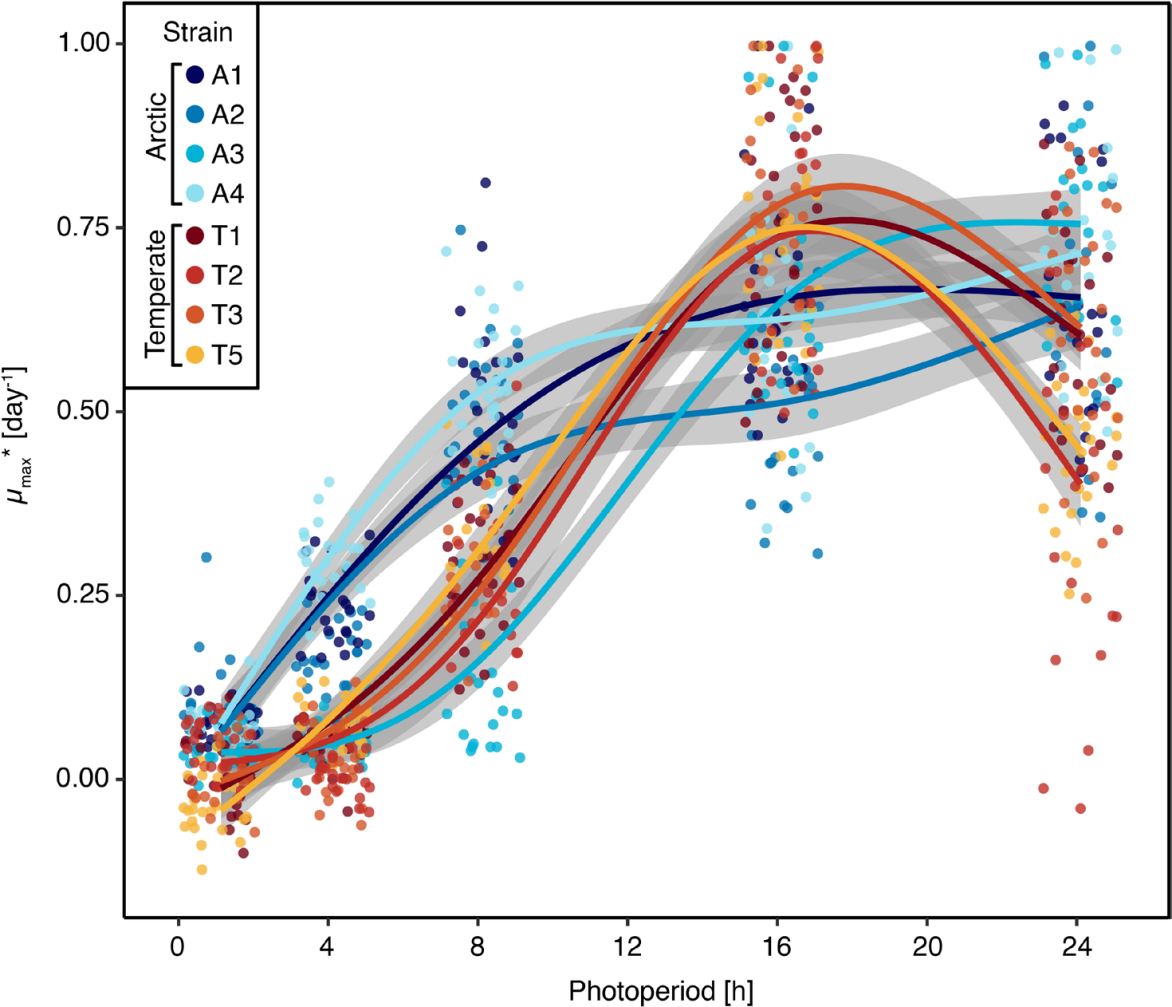
Photoperiod reaction norms of 4 strains of *T. gravida* (A1–A4) and *T. rotula* (T1–T3; T5), respectively, with normalized maximum growth rate on the *y*‐axis (*μ*_max_*) and applied photoperiod (in hours) on the *x*‐axis. GAMs ± SE are fitted to the data points for each strain. For visibility, *x*‐axis jittering was added to the points (GAMs were fit to unjittered data).

The photoperiod reaction norms of the tested Arctic and temperate diatom strains displayed distinct shapes that differed significantly (*p* < 0.001, Table S3) between the species, that is, between Arctic and temperate origin (Figure 2). The reaction norm for the Arctic strains reached the highest growth rates at photoperiods of 24 h. During the initial phase of the reaction norm, the growth rates of strain A1, A2, and A4 increased with photoperiod following a saturation function, while strain A3 followed a sigmoidal shape. Contrastingly, the photoperiod reaction norms of the temperate strains showed a sigmoidal shape with an optimum photoperiod of 17.3 ± 0.2 h, after which growth decreased again. To summarize, the growth performance incline of Arctic diatoms was steeper at short photoperiods compared to temperate diatoms and showed no decrease at 24 h photoperiods, whereas the growth performance of temperate diatoms deteriorated at 24 h photoperiods.

### 3.3 Nitrate Uptake Rates

**FIGURE 3:**
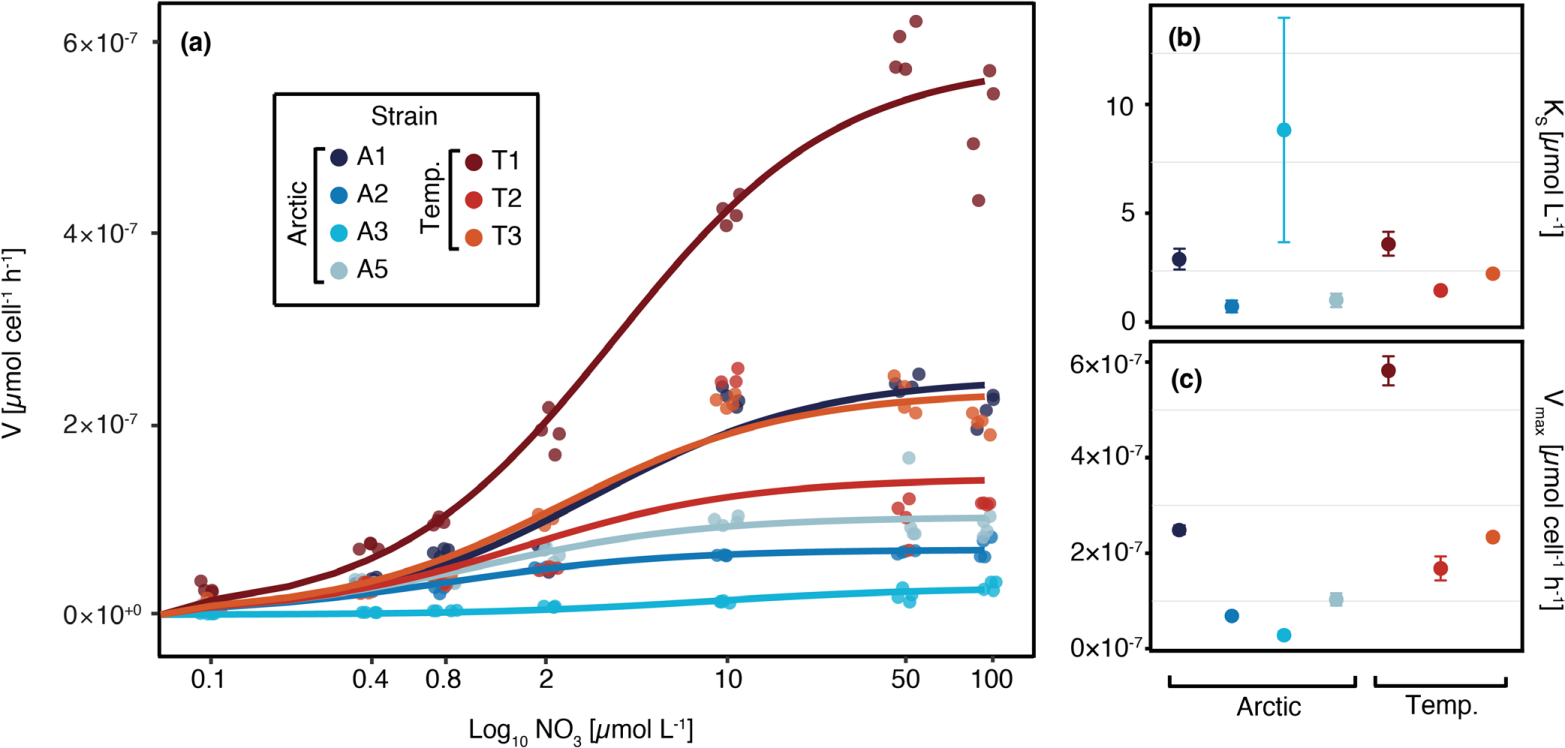
Nitrate uptake kinetics and associated parameters for *T. gravida* (strains A1, A2, A3, and A5) and *T. rotula* (strains T1, T2, T3) indicated by color with (a) cell‐normalized nitrate uptake rates across the tested nitrate concentrations fitted to the Michaelis–Menten function plotted on a logarithmic *x*‐axis; (b) half‐saturation constant (*K*_*S*_); and (c) maximum uptake rate (*V*_max_). Uptake parameters (panels b and c) are given as mean ± SD. Note that uptake experiments for Arctic and temperate strains had to be performed at different temperatures (9°C vs. 16°C, see Section 2.4) and so may not be directly comparable.

The maximum uptake rates (*V*_max_) for nitrate revealed a wide range among the Arctic and temperate strains (Figure 3). Precisely, *V*_max_ ranged from 0.29 ± 0.08 × 10^−7^ μmol N cell^−1^ h^−1^ in the Arctic strain A3 up to 5.82 ± 0.30 × 10^−7^ μmol N cell^−1^ h^−1^ in the temperate strain T1. Despite generally higher *V*_max_ values for most temperate diatom strains, we did not find strong evidence for differences between latitudes (*p* = 0.14). Similarly, half‐saturation constants (*K*_*S*_) showed high intraspecific variability, particularly in strain A3, resulting in no significant differences (*p* = 0.70). Overall, nitrate uptake‐related traits did not reveal significant differences between the studied Arctic and temperate diatoms.

### 3.4 Convergent Amino Acid Substitutions

**FIGURE 4:**
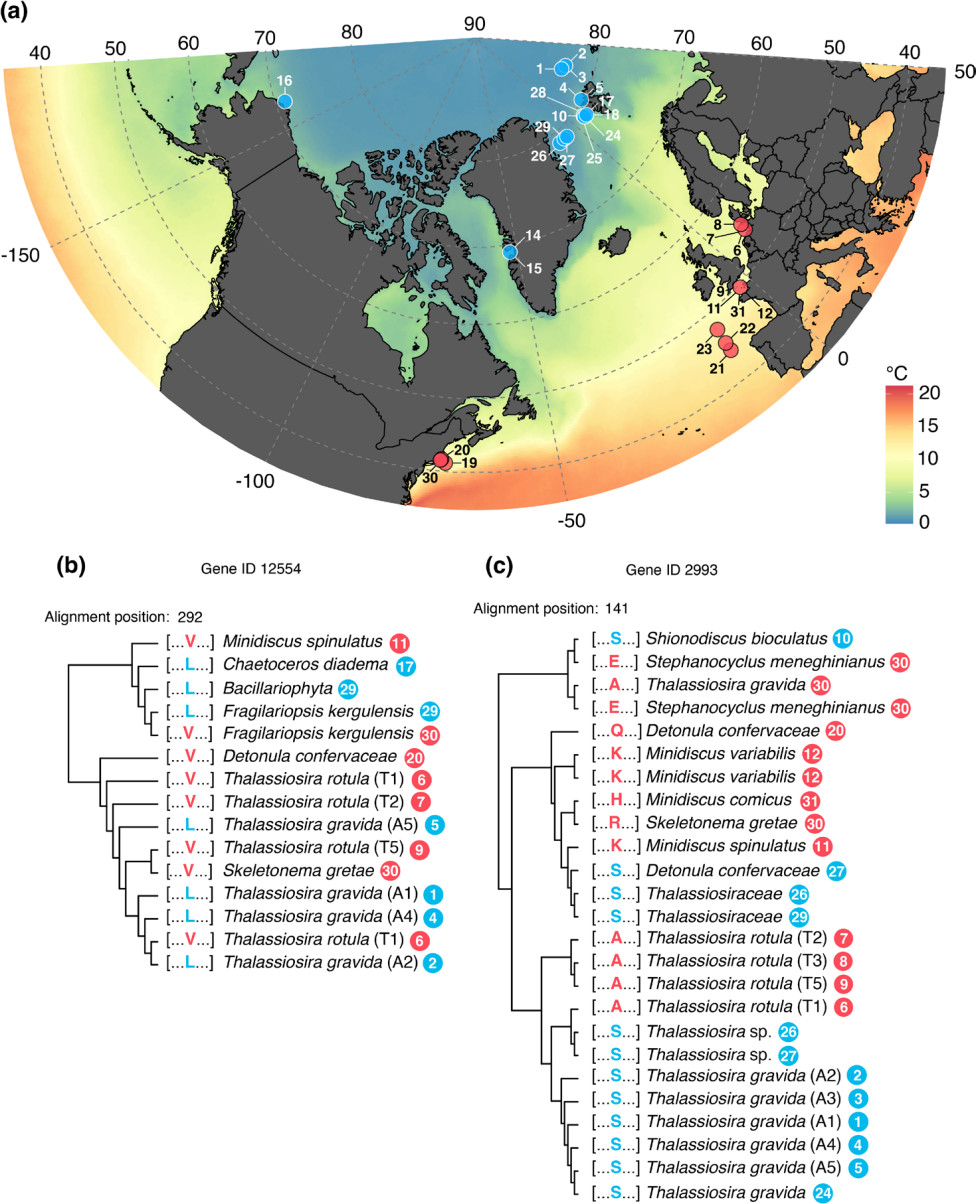
Map of origin for metatranscriptomes and culture isolate RNA‐seq datasets used in the CAAS analysis (see details and references for datasets in Table S2), colored by annual mean sea surface temperature (modified after NASA 2014). Map lines delineate study areas and do not necessarily depict accepted national boundaries. RNA‐seq datasets from Arctic and temperate ocean provinces are marked as blue and red dots, respectively and are numbered with a location code (a). Exemplary midpoint rooted phylogenetic trees of two candidate genes resulting from the CAAS analysis are shown for gene ID 12554 with habitat‐specific amino acids in Arctic and temperate diatoms at alignment position 292 (b), and gene ID 2993 with CAAS only in Arctic diatoms at alignment position 141 while showing diverse amino acids in temperate diatoms (c). Phylogenetic trees highlight phylogenetic independence of the found CAAS. Tips are labeled with the respective amino acid (one‐letter code) at the corresponding CAAS site, the assigned taxonomy, and the location code of the respective RNA‐seq data set. CAAS and station code are colored blue or red for Arctic or temperate origin, respectively.

After quality filtering, we identified 26 candidate gene alignments with specific CAAS, that is, convergence towards a specific amino acid at a specific alignment position as a response to Arctic or temperate origin of the sequence (Figure 4, Table 2). In total, 12 of these candidate gene alignments could be functionally annotated. For convenience, members of each candidate gene alignment are collectively called “IDs” in the following sections. Of the 12 functionally annotated IDs, 6 IDs are annotated as being involved in the central dogma processes (i.e., fundamental processes of the flow of genetic information: DNA replication, transcription, and translation that are responsible for the maintenance and expression of genes within an organism). A further 5 IDs are annotated as being involved in metabolic functions, including the often interrelated functions of lipid‐ and secondary metabolism. Among all 26 identified IDs, 22 of those exclusively contained CAAS in the Arctic diatom sequences (i.e., one unique AA shared between all identified Arctic diatom sequences, while temperate sequences show different AAs at the respective alignment position, e.g., Figure 4c). For 3 IDs, CAAS were identified only in temperate sequences, and 1 ID showed a CAAS for both origins simultaneously (i.e., one unique AA for Arctic and temperate sequence, respectively, at the same alignment position, Figure 4b). With regard to taxonomic coverage, the Arctic‐origin CAAS with the highest number of diatom species was found in a sugar transporter (gene ID 7700) shared among *T. gravida* , *Thalassiosira oceanica*, *Shionodiscus bioculatus*, and *Detonula confervaceae*. For the temperate CAAS, the highest taxonomic coverage was found in one of the zinc finger gene families (Sec23/Sec24, gene ID 6123) shared across seven diatom species ( *T. rotula* , *Minidiscus spinulatus*, *Minidiscus variabilis*, *Minidiscus comicus*, *Detonula confervaceae*, *Leptocylindrus danicus* , *Chaetoceros* sp.). Besides identifying specific gene candidates potentially carrying habitat‐specific adaptations, the CAAS analysis demonstrated a higher abundance of these convergent molecular adaptations in Arctic diatoms compared to temperate diatoms.

**TABLE 2:** Results of CAAS analysis. For each alignment ID reported by CAAStools that passed quality filtering and manual quality control, alignment position (Pos.), amino acid of Arctic sequences (Arctic AA) and temperate sequences (Temperate AA) as well as the functional annotation from the eggNOG‐mapper results are given.

Alignment ID	Pos.	Arctic AA	Temperate AA	Functional annotation	Functional group
9895	384	N	D, F, K, T	Methylation of tRNAs	[J] Translation, ribosomal structure and biogenesis
1375	52	S	A, D, P, V	Zinc finger, C3HC4 type	[K] Transcription
2720	413	N	Q, S	Zinc finger, Nab2‐type	[K] Transcription
6123	30	A, I	V	Sec23/Sec24 zinc finger	[K] Transcription
2998	749	E	C, G, I, Q, R, S, T	DNA mismatch repair protein Mlh1	[L] Replication, recombination and repair
7630	448	E	K, Q, S	DNA polymerase alpha/epsilon subunit B	[L] Replication, recombination and repair
7700	423	V	E, G, S, T	Sugar transporter	[G] Carbohydrate transport and metabolism
5037	269	Q	E, K, R	Membrane‐bound acyltransferase	[I] Lipid transport and metabolism
5781	218	K	E, G, S, V	Oxidation–reduction (dehydrogense)	[I] Lipid transport and metabolism and [Q] Secondary metabolites biosynthesis, transport and catabolism
6653	168	E	D, G, Q	All‐trans‐retinol 13,14‐reductase	[I] Lipid transport and metabolism and [Q] Secondary metabolites biosynthesis, transport and catabolism
8045	893	S	A, V	ABC transporter	[Q] Secondary metabolites biosynthesis, transport and catabolism
8045	1162	V	A, I		
8045	1259	A	I, K, V		
631	616	W	A, F, H, L, Y	Chitinase class I	[R] General function prediction only
557	16	L	A, F, I, S, T	—	[S] Function unknown
908	158	D, F, T	S	—	[S] Function unknown
2702	371	C	F, S, Y	—	[S] Function unknown
2993	141	S	A, E, H, K, Q, R	—	[S] Function unknown
3485	150	S	A, D, E, T	—	[S] Function unknown
4072	662	M	A, G, L, P, S, T, V	—	[S] Function unknown
4756	49	A	E, N, T, V	—	[S] Function unknown
4990	33	E	A, D, K, N, Q, S, T, V	—	[S] Function unknown
6457	27	A	K, P, S	—	[S] Function unknown
10130	156	N	D, F, G, L, S	—	[S] Function unknown
11282	276	N	K, L, R, V	—	[S] Function unknown
11341	215	M	I, K, L, S	—	[S] Function unknown
12554	333	L	V	—	[S] Function unknown
13375	292	A, F, I	V	—	[S] Function unknown

## 4 Discussion

The Arctic and temperate diatom strains showed distinct temperature reaction norms with significant differences in *T*_opt_ and thermal breadth but no significant differences in *E*_*A*_ and *μ*_max_. With regard to photoperiod‐dependent growth, reaction norm shapes varied between short photoperiods and diverged at 24 h of constant light for Arctic and temperate strains. Large trait variability was observed in nitrate uptake parameters *V*_max_ and *K*_*S*_. In terms of genetic traits, a screening of available (meta‐) transcriptomes for CAAS revealed 26 candidate genes pointing to habitat‐specific molecular adaptations, most of which were found in Arctic diatoms. The CAAS‐bearing proteins with functional annotations were dominated by central dogma processes, followed by functions in lipid and secondary metabolism.

### 4.1 Thermal Traits Indicate a Potential for Poleward Range Shifts of Temperate Diatoms

The *T*_opt_ of the Arctic and temperate diatom strains differed according to their thermal habitats. While a *T*_opt_ of 18.4°C lies at the upper range of the temperate diatoms' habitat (Wiltshire and Manly 2004), a *T*_opt_ of around 10°C for the Arctic diatoms seems surprisingly high, as such water temperatures are neither reached nor surpassed in the Arctic Ocean (Timmermans and Labe 2022). However, this seemingly high *T*_opt_ can buffer the Arctic diatoms against temperature fluctuations and can prevent detrimental interactive effects induced under resource limitation that effectively lower *T*_opt_ (Edwards et al. 2016; Thomas et al. 2017). Habitat‐specific adaptation can also be observed in differences in the thermal breadth of the TPCs and correspond to the eurytherm and stenotherm lifestyles imposed by the temperature amplitudes of Arctic and temperate habitats. The temperate diatom strains, which are subject to larger annual temperature variation (Wiltshire and Manly 2004) hence exhibited broader TPCs compared to the narrower thermal breadth of the Arctic strains. No origin‐dependent differences were found for *E*_*A*_ and *μ*_max_, contradictory to the Eppley curve that predicts exponentially increasing *μ*_max_ values with increasing *T*_opt_ (Kremer et al. 2017). This indicates biochemical adjustments in the cellular machinery of the Arctic diatoms that effectively lower their thermodynamic constraints (Barton and Yvon‐Durocher 2019; Liu et al. 2021). Furthermore, a recent meta‐analysis and laboratory experiments suggest that *μ*_max_ is better explained by phylogeny than latitude (Kontopoulos et al. 2020; Rehder et al. 2024), in congruence with the similar maximum growth rates at different *T*_opt_ observed in our study.

With regard to poleward migration potential, the thermal range of the temperate diatom strains would allow them to tolerate Arctic temperatures, but at a competitive disadvantage in terms of growth rates. However, the known interactive effects of temperature‐dependent growth with nitrate and light intensity (Edwards et al. 2016; Thomas et al. 2017) may alleviate this competitive disadvantage. Precisely, in a scenario of elevated temperatures in combination with resource limitation (Steinacher et al. 2010), the Arctic diatom strains may be pushed beyond their *T*_opt_, while the temperate diatom strains likely would grow closer to their respective *T*_opt_ (Thomas et al. 2017).

### 4.2 Extreme Photoperiods at High Latitudes May Pose a Barrier to the Arctic

The photoperiod reaction norms assessed in this study showed habitat‐specific adaptations that reflect an imprint of a chronobiological background that is strongly conserved even after years of laboratory cultivation at a constant intermediate photoperiod (Giesler et al. 2023). In line, the response of the tested conditions in this study showed to be reproducible across strains from the same origin and also had comparable responses at the respective experimental conditions in the study by Giesler et al. (2023). While the temperate strains displayed a photoperiod optimum of approximately 17 h, mirroring a habitat characterized by intermediate daylengths, the Arctic strains showed specific competitive advantages at both extremely long and extremely short photoperiods. This response is likely a consequence of polar day and polar night. In contrast to the light regimes of temperate regions, intermediate photoperiods only correspond to a transitional phase lasting a few weeks in the high Arctic.

On the cellular level, prolonged photoperiods and the absence of darkness periods enhance the excitation pressure on Photosystem II (PSII) (Lepetit et al. 2017, 2012), hinder photodamaged PSII repair in darkness (Xu et al. 2017), and cause an increase in oxidative stress (Roeber et al. 2021). Arctic diatoms have been shown to combat those conditions by fine‐tuning specific adaptations in terms of their photosynthetic machinery (Croteau et al. 2020), specifically by mechanisms that allow for effective light energy dissipation and reactive oxygen species (ROS) scavenging rather than enhancing non‐photochemical quenching (NPQ) (Guérin et al. 2024). Moreover, enhanced ROS scavenging was also shown to arise from symbiotic interactions with associated bacteria (Hunken et al. 2008).

The decreased growth performance for temperate diatoms at a 24 h photoperiod compared to Arctic diatoms suggests the lack of these adaptations. As a consequence, extreme photoperiods in polar regions may act as a barrier, slowing the rate of poleward range shifts of temperate diatoms (Huffeldt 2020; Tougeron 2021). In fact, a study by Chivers et al. (2017) found that dinoflagellates follow poleward isotherm shifts more closely than temperate diatoms. Such observations indicate that photoautotrophs are particularly constrained by the polar light regime as they depend on photosynthetic performance to maintain their competitiveness (Tittel et al. 2003). This underlines the need for further studies to investigate whether temperate diatoms can adapt to polar photoperiods and, if so, whether there are trade‐offs associated with this. As a result of adaptation to other drivers such as temperature, nutrient limitation, and ocean acidification, the existence of such trade‐offs has already been confirmed for phytoplankton (Aranguren‐Gassis et al. 2019; Jin, Ji et al. 2022; O'Donnell et al. 2018). Associated costs of the respective trade‐offs include impaired energy production (Aranguren‐Gassis et al. 2019; Jin, Ji et al. 2022; O'Donnell et al. 2018), lowered nutrient affinity (O'Donnell et al. 2018), or the loss of genetic diversity (Jin, Wan et al. 2022). Given that the Arctic diatom strains experienced a constant photoperiod of 14:10 h light:dark during long‐term cultivation in the culture collection (Table S1) and still maintain a strong adaptive signal to extreme Arctic photoperiods, this suggests that photoperiodic adaptation is likely to be costly and a rather long‐term process.

### 4.3 Large Variability Indicates Low Selective Pressure on Nitrate Uptake‐Related Traits

The maximum uptake rate of nitrate as well as the half saturation constant showed high variability across the tested diatom strains. Yet it has to be considered how the results are affected by the different temperatures at which the assays had to be conducted for Arctic and temperate diatoms. Previous studies demonstrated that an organism's nitrate affinity decreases at temperatures below its optimum (Nedwell 1999; Reay et al. 1999). Therefore, direct comparisons between the determined nitrate uptake traits and resulting implications for competitive advantages are complicated. Nevertheless, the observed large intraspecific variability suggests a relatively low selective pressure on nitrate uptake rates in temperate and Arctic habitats. Indeed, environmental nitrate concentrations in Arctic and temperate habitats show annual variation from near zero concentrations in the late summer months up to approximately 10 and 20 μmol L^−1^ during winter/early spring in northeast Svalbard (Arctic) and Helgoland (North Sea), respectively (Randelhoff et al. 2015; Wiltshire 2011). Thus, due to trade‐offs of nutrient uptake with other metabolic processes (Ward et al. 2016), it might be beneficial to maintain high variability in nutrient uptake‐related traits in a population to quickly respond to nutrient fluctuations (Raimbault et al. 1990). To maintain this variability in the context of poleward range shifts, the temperate diatoms membrane lipids and uptake transporters likely require specific molecular adaptations as their flexibility and effectiveness decrease with temperature (Nedwell 1999). The presence of these habitat‐specific adaptations in Arctic diatoms is indicated by the CAAS analysis in which multiple transporters and lipid‐related gene candidates with molecular adaptations were identified (see below).

### 4.4 Abundant CAAS in Arctic Diatoms Indicates Further Habitat‐Specific Adaptations

By analysing temperate and Arctic diatom transcriptomes and (meta‐) transcriptomes, 26 proteins with habitat‐specific CAAS could be identified. These molecular adaptations likely contribute to the found differences in diatom adaptation to different thermal and light regimes. Most of the CAAS showed selection towards an identical amino acid in Arctic diatoms, while the temperate diatoms had variable amino acids at the respective alignment position. These molecular adaptations indicate hard selection pressure favoring the fixation of these substitutions in Arctic populations and species. Consequently, the resulting traits of these adaptations likely facilitate their distribution in Arctic habitats (Birkeland et al. 2020; Tong 2024).

The majority of the CAAS‐bearing proteins are involved in central dogma processes that transfer genetic information within cells. To some extent, it is reasonable to assume that thermal differences in the respective habitats may have contributed to the natural selection of these CAAS, coordinating transcription and translation to ensure cellular homeostasis in coherence with further environmental factors (Knapp and Huang 2022). In this context, the convergent modulation of zinc finger transcription factor sequences could potentially contribute to cellular homeostasis by, for example, altering their binding affinities to promoter regions (Knapp and Huang 2022). Functionally, the identified zinc finger transcription factors are known to be involved in abiotic stress responses in plants (Han et al. 2022) and the regulation of growth and photosynthesis, particularly in polar diatoms (Ye et al. 2022). The CAAS discovered in DNA polymerase and DNA mismatch repair proteins also suggest a functionally coherent parallel adaptation of these related processes (i.e., synthesizing nucleotide strands and correcting for potential errors). The discovery of CAAS in a membrane‐bound acyltransferase and in the all‐trans‐retinol 13,14‐reductase suggests additional adaptations and merits further investigation. In general, membrane‐bound acyltransferases are involved in the modification and synthesis of complex membrane lipids, which previously have been associated with thermal adaptation (Liang et al. 2019; Nedwell 1999; Svenning et al. 2024). The CAAS in all‐trans‐retinol 13,14‐reductase may contribute to photobiological regulation, as it can play a role in pathways that supply both the chromophore for rhodopsin photoreceptors and molecules involved in photoprotection in diatoms, thus contributing to photoreception and cellular energy regulation (Dong et al. 2016; Marchetti et al. 2012).

We note that many of the identified CAAS‐bearing proteins could not be functionally annotated, leaving their specific contribution to the polar adaptation of diatoms open. This highlights a crucial gap in our understanding of the ecologically important functions of diatoms and constraints assessing the potential consequences and possibilities of eventual range shifts. Indeed, the functional complexity of the proteins in which CAAS are detected raises questions about the extent to which the migration and long‐term persistence of temperate *Thalassiosira* populations in the Arctic are possible in response to immediate or current global change conditions. Although the degree to which a respective CAAS is essential for a specific diatom population to establish in an Arctic habitat may differ among the identified proteins, the sum of habitat‐specific functional genetic modifications represents potential bottlenecks of adaptation that can hamper poleward migration of temperate diatoms. For example, adaptations to polar light regimes are reflected not only in the photoperiod reaction norms but potentially also in transcription factors regulating photosynthesis and growth, as well as in enzymes crucial for photobiological regulation. Yet, as a consequence of ocean warming, CAAS that are the result of thermal adaptation may become less important while genetic adaptations to more stable physical parameters unique to Arctic latitudes, such as the extreme light regimes, will prevail.

## 5 Conclusion

This study highlights different facets of Arctic adaptation. These include adaptations to polar photoperiods and temperatures that are shaped by molecular adaptations that ensure information flow from DNA to proteins, and molecular adaptations within lipid, carbohydrate, and secondary metabolism. The assessed thermal traits indicate the potential for poleward range shifts of temperate diatoms in response to the ongoing warming of the Arctic, but the different photoperiod response norms highlight barriers that are not yet considered in species distribution models. In addition, the identified candidate genes in which adaptation to Arctic habitats is most evident provide a first comparative insight into the convergent evolution of Arctic diatoms, but also underline our lack of knowledge of ecologically important gene functions governing polar adaptation. Finally, our results open new targets for further studies to investigate the consequences of global warming on marine primary producers and to estimate adaptation speed under polar day scenarios to gain a more realistic understanding of poleward range shifts.

## Author Contributions

**Jakob K. Giesler:** conceptualization, data curation, formal analysis, investigation, methodology, software, validation, visualization, writing – original draft, writing – review and editing. **Dedmer B. Van de Waal:** data curation, methodology, resources, validation, writing – review and editing. **Mridul K. Thomas:** conceptualization, methodology, validation, writing – review and editing. **Luka Šupraha:** data curation, methodology, software, supervision, writing – review and editing. **Florian Koch:** data curation, formal analysis, methodology, validation, writing – review and editing. **Tilmann Harder:** conceptualization, funding acquisition, methodology, project administration, supervision, writing – review and editing. **Carla M. Pein:** investigation, methodology, validation, writing – review and editing. **Uwe John:** funding acquisition, project administration, resources, writing – review and editing. **Sylke Wohlrab:** conceptualization, formal analysis, funding acquisition, investigation, methodology, project administration, supervision, writing – original draft, writing – review and editing.

## Conflicts of Interest

The authors declare no conflicts of interest.

## Supporting information


Data S1.

## Data Availability

The data that support the findings of this study are openly available in the PANGAEA data repository at https://doi.org/10.1594/PANGAEA.972918 (thermal reaction norm data), https://doi.org/10.1594/PANGAEA.972917 (photoperiod reaction norm data), and https://doi.org/10.1594/PANGAEA.972919 (nitrate uptake data). RNA‐seq data produced in this study have been deposited in the European nucleotide archive (ENA) under the project accession number PRJEB78462. Sea surface temperature data were obtained from the UNEP World Conservation Monitoring Centre at https://doi.org/10.34892/tf0n‐6t02.
