# Supplementary material for: What Does It Mean to Be(Come) Arctic? Functional and Genetic Traits of Arctic‐ and Temperate‐Adapted Diatoms
Source: Glob Chang Biol. 2025 Mar 20;31(3):e70137. doi: 10.1111/gcb.70137 (PMC11924310; doi:10.1111/gcb.70137)

# What does it mean to be(come) Arctic? Functional and genomic traits of Arctic and temperate adapted diatoms

## Supplementary Material

**Table S1:** Detailed available information about the diatom strains used in this study. Information contains species name, strain name as used throughout the manuscript (ID), the official strain name as used in the respective culture collections where the strains originated from (strain name), isolation date, and the sampling location (latitude, longitude) in decimal degrees (if available). Moreover, culture conditions during long-term cultivation in the respective culture collections are given, showing that both Arctic and temperate strains were kept at rather intermediate photoperiods before they were obtained for this study.

| species | ID | Strain name | isolation date | latitude | longitude | culture collection temperature | culture collection photoperiod |
|---|---|---|---|---|---|---|---|
| *T. gravida* | A1 | UiO478 | 2018-08-20 | 83.33 | 29.29 | 4°C | 14:10h |
| *T. gravida* | A2 | UiO483 | 2018-08-05 | 83.15 | 31.46 | 4°C | 14:10h |
| *T. gravida* | A3 | UiO484 | 2018-08-05 | 83.15 | 31.46 | 4°C | 14:10h |
| *T. gravida* | A4 | UIO447 | 2017-09-12 | 79.99 | 14.97 | 4°C | 14:10h |
| *T. gravida* | A5 | UiO448 | 2017-09-12 | 79.99 | 14.97 | 4°C | 14:10h |
| *T. rotula* | T1 | t_rotula_S16 | unknown | 54.183333 | 7.9 | 15°C | 16:8h |
| *T. rotula* | T2 | strain_WHV | 2022-03-06 | 54.183333 | 7.9 | 15°C | 16:8h |
| *T. rotula* | T3 | strain_Sylt | 2021-09-01 | 55.0300 | 8.46 | 15°C | 16:8h |
| *T. rotula* | T4 | RCC_778 | unknown | English channel | | 13°C | 12:12h |
| *T. rotula* | T5 | RCC_290 | unknown | English channel | | 20°C | 12:12h |

**Table S2:** Details and references for RNA-seq input data for CAAStools analysis. For each RNA-seq data set location code number in Figure 4 of the main manuscript is given, as well as RNA-seq type (i.e., obtained from culture isolate or metatranscriptomic field sample), species and strain (in case of culture isolates), origin and specific sampling or isolation coordinates and data references. Coordinates marked with an asterisk (*) indicate approxim ate coordinates as exact location could not be identified.

| Location Code (Fig.4) | RNA-seq type | Species | Strain | Origin | Lat | Long | Reference |
|---|---|---|---|---|---|---|---|
| 1 | Isolate | Thalassiosira gravida | NORCCA UiO478 (A1) | Arctic | 83.33 | 29.29 | this study |
| 2 | Isolate | Thalassiosira gravida | NORCCA UiO483 (A2) | Arctic | 83.15 | 31.46 | this study |
| 3 | Isolate | Thalassiosira gravida | NORCCA UiO484 (A3) | Arctic | 83.15 | 31.46 | this study |
| 4 | Isolate | Thalassiosira gravida | NORCCA UiO447 (A4) | Arctic | 79.99 | 14.97 | this study |
| 5 | Isolate | Thalassiosira gravida | NORCCA UiO448 (A5) | Arctic | 79.99 | 14.97 | this study |
| 6 | Isolate | Thalassiosira rotula | Harder Lab t.rotula S16 (T1) | Temperate | 54.18 | 7.90 | this study |
| 7 | Isolate | Thalassiosira rotula | WHV (T2) | Temperate | 54.18 | 7.90 | this study |
| 8 | Isolate | Thalassiosira rotula | Sylt (T3) | Temperate | 55.03 | 8.46 | this study |
| 9 | Isolate | Thalassiosira rotula | RCC290 (T4) | Temperate | 49.79* | -2.57* | this study |
| 10 | Isolate | Shionodiscus bioculatus | - | Arctic | 78.98 | 9.48 | this study |
| 11 | Isolate | Minidiscus spinulatus | RCC4659 | Temperate | 49.79* | -2.57* | https://zenodo.org/records/5256603 |
| 12 | Isolate | Minidiscus variabilis | RCC4665 | Temperate | 49.79* | -2.57* | https://zenodo.org/records/5256603 |
| 13 | Isolate | Minutocellus polymorphus | - | Temperate | -17.50 | 11.25 | https://evocellbio.com/eukprot/ |
| 14 | Isolate | Fragilariopsis sp. | - | Arctic | 69.07 | -52.41 | https://doi.org/10.1186/s12867-019-0124-0 |
| 15 | Isolate | Pseudo-Nitzschia turgidula | - | Arctic | 69.07 | -52.41 | https://doi.org/10.1186/s12867-019-0124-0 |
| 16 | Isolate | Arcocellulus cornucervis | - | Arctic | 71.18 | -159.42 | https://evocellbio.com/eukprot/ |
| 17 | Isolate | Chaetoceros diadema | - | Arctic | 78.98 | 9.48 | this study |
| 18 | Isolate | Pseudo-Nitzschia turgidula | - | Arctic | 78.98 | 9.48 | this study |
| 19 | Isolate | Dactyliosolen fragilissimus | - | Temperate | 41.33 | -70.57 | https://evocellbio.com/eukprot/ |
| 20 | Isolate | Detonula confervaceae | - | Temperate | 41.60 | -71.40 | https://evocellbio.com/eukprot/ |
| 21 | Metatranscriptome | - | - | Temperate | 44.28 | -12.61 | DOI: 10.46936/10.25585/60000951 |
| 22 | Metatranscriptome | - | - | Temperate | 45.53 | -12.43 | DOI: 10.46936/10.25585/60000951 |
| 23 | Metatranscriptome | - | - | Temperate | 47.57 | -12.11 | DOI: 10.46936/10.25585/60000951 |
| 24 | Metatranscriptome | - | - | Arctic | 78.85 | 9.23 | DOI: 10.46936/10.25585/60000951 |
| 25 | Metatranscriptome | - | - | Arctic | 78.87 | 8.11 | DOI: 10.46936/10.25585/60000951 |
| 26 | Metatranscriptome | - | - | Arctic | 79.02 | -9.52 | DOI: 10.46936/10.25585/60000951 |
| 27 | Metatranscriptome | - | - | Arctic | 79.04 | -7.67 | DOI: 10.46936/10.25585/60000951 |
| 28 | Metatranscriptome | - | - | Arctic | 79.07 | 7.08 | DOI: 10.46936/10.25585/60000951 |
| 29 | Metatranscriptome | - | - | Arctic | 79.08 | -8.52 | DOI: 10.46936/10.25585/60000951 |
| 30 | Metatranscriptome | - | - | Temperate | 41.50 | -71.33 | https://doi.org/10.1073/pnas.1421993112 |
| 31 | Isolate | Minidiscus comicus | | Temperate | 49.79* | -2.57* | https://zenodo.org/records/5256603 |

**Table S3:** ANOVA results testing for differences of GAM shape as a response of photoperiod and the interaction of photoperiod and strain origin. Reference degrees of freedom (Ref. df), F-, and p-values are reported for each effect. Values marked with an asterisk (*) indicate significant effects (p < 0.05).

| | | GAM shape | | |
|---|---|---|---|---|
| | *Ref. df* | *F* | *p* | |
| photoperiod | 3 | 420.53 | <0.001 | * |
| photoperiod*origin | 3 | 49.96 | <0.001 | * |

**Figure S1:** Phylogenetic tree of ITS1 sequences for the *T. rotula* and *T. gravida* strains used in this study and respective reference sequences for both species (strains JX074…) identified by Whittaker et al (2012).

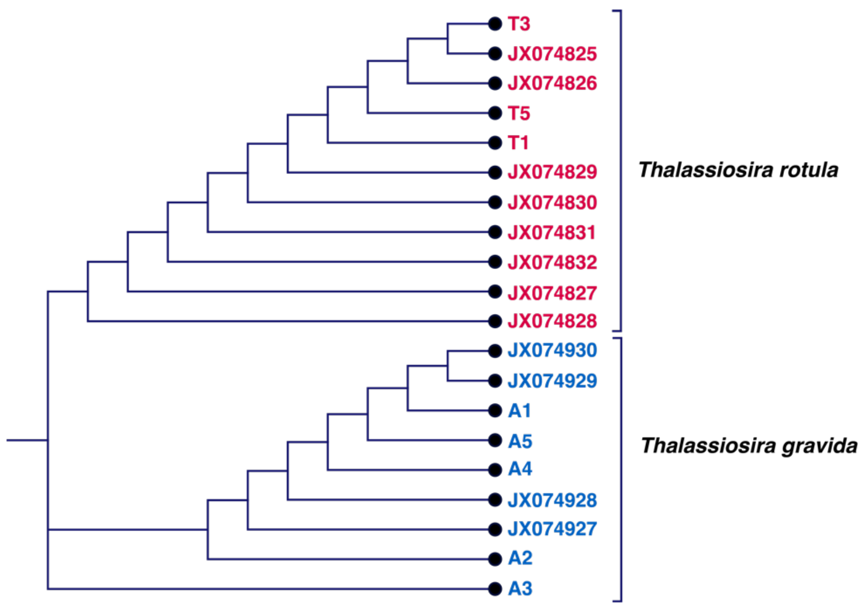

Supplement: Supplementary file 1 — Data S1. [file GCB-31-e70137-s001.pdf]
